# Biochemical and clinical markers of endothelial dysfunction do not outweigh traditional risk factors for the presence of diabetic retinopathy in patients with type 1 diabetes

**DOI:** 10.1186/s13098-022-00912-y

**Published:** 2022-09-27

**Authors:** Alessandra Saldanha de Mattos Matheus, Maria de Fátima Bevilacqua da Matta, Eliete Leão Silva Clemente, Maria de Lourdes Guimarães Rodrigues, Débora Cristina Torres Valença, Karla Rezende Guerra Drummond, Marília Brito Gomes

**Affiliations:** 1 Diabetes Unit, State University of Rio de Janeiro, Boulevard 28 de Setembro, n. 77–Vila Isabel, Rio de Janeiro, 20551-030 Brazil; 2 Clinical and Experimental Physiopathology of Hypertension Unit, State University of Rio de Janeiro, Rio de Janeiro, Brazil

**Keywords:** EndoPAT, Diabetes type 1, Microvascular reactivity, Biochemical markers, Diabetic retinopathy

## Abstract

**Background:**

This study aimed to evaluate whether soluble vascular cytoadhesive molecule-1 (sVCAM-1), intracellular cytoadhesive molecule-1 (sICAM-1), and endothelial function as assessed by EndoPat outweighed traditional risk factors for the presence of diabetic retinopathy (DR) in patients with type 1 diabetes (T1D).

**Methods:**

Patients aged ≥ 12 years completed a clinical–epidemiological questionnaire. Fasting venous blood samples were obtained (lipid profile, glycemic control, and C-reactive protein levels). Vascular reactivity was assessed via peripheral arterial tonometry performed by supplying the reactive hyperemia index (RHI) through the EndoPAT device. sVCAM-1 and sICAM-1 levels were measured using multiplex assays.

**Results:**

Data were obtained from 187 patients (51.3% female), aged 32 ± 13 years with a disease duration of 14 (6–15) years and mean hemoglobin A1c (HbA1c) of 9.1% ± 2.1%. After adjustments were made, age, HbA1c, arterial blood pressure, and use of drugs that could interfere with endothelial function were found to be associated with DR. No association was noted with sVCAM-1 and sICAM-1 levels and RHI.

**Conclusions:**

In our sample, sVCAM-1, sICAM and EndoPAT did not outweigh the traditional DR risk factors, such as age, high HbA1c, arterial blood pressure, and use of drugs that could interfere with endothelial function and are significantly associated with DR. Further prospective studies should evaluate if markers of endothelial dysfunction could predict diabetes-related micro and macrovascular complications in T1D.

## Background

Type 1 diabetes (T1D) has long been associated with chronic micro- and macro-vascular complications, such as diabetic retinopathy (DR), chronic diabetic renal disease, neuropathy, and cardiovascular diseases (CVDs). So far, the most important traditional risk factors related to these complications were found to be age, duration of diabetes, hemoglobin A1c (HbA1c) and blood pressure as described in the *Diabetes Control and Complications Trial/Epidemiology Of Diabetes Intervention And Complications* (DCCT/EDIC) [1]. Besides the previously mentioned risk factors, early endothelial dysfunction (ED) has also been linked to microvascular complications [2, 3]. High levels of advanced glycosylation end products (AGEP), which are produced by chronic hyperglycemia and oxidative stress, lead to a reduction in the bioavailabiliy of nitric oxide from the endothelium that in turn leads to an increase in the expression of vascular and intercellular adhesion molecules. This process allows for leukocytes to attach to the endothelial surface and promote inflammation [4].

Among adhesion molecules, soluble vascular cytoadhesive molecule-1 (sVCAM-1) and intracellular cytoadhesive molecule-1 (sICAM-1) are the most extensively studied and have an important role during early stages of vascular complications in T1D [5, 6]. Both are inflammation-related biochemical markers and are associated with ED (3). The *Diabetes Control and Complications Trial* (DCCT) [7] was the first study to demonstrate strong association between inflammatory activity with these biochemical markers (sICAM-1 and sVCAM-1). However, both molecules play a different role in the pathophysiology of microvascular complications. For instance, elevated levels of sVCAM-1 are found in young people with T1D, are related to metabolic decompensation and a family history of cardiovascular diseases [8], and seem to be an important factor for retinopathy development [9, 10]. However, elevated levels of sICAM-1 are associated with the presence of albuminuria [11] and neuropathy [12]. High levels of sICAM were found in patients with recent-onset T1D [6] without the presence of macrovascular complications [5]. Thus, these findings suggest that soluble cell adhesion molecules could play an important role in diagnosis and prognosis of microvascular diabetic complications [13].

Besides biochemical markers, ED can be assessed using several techniques that are used to detect early pro-atherogenic process [14]. EndoPAT uses the reactive hyperemia index (RHI) and is a non-invasive and practical method showing scores that distinguish those patients with Type 2 Diabetes (T2D) with high cardiovascular risk (CVR) from those with low CVR risk. In T1D, this method could also identify endothelial function impairment when compared to healthy controls. Haller et al. [15] studied 44 children with T1D and found lower RHI values compared to children without diabetes with an average coefficient variation of 14.8%. When compared with healthy controls, Mahmud et al. [16] concluded that male adolescents with T1D presented ED as assessed by EndoPAT.

The present study aimed to evaluate whether sVCAM-1, sICAM-1, and endothelial function assessed by EndoPAT in patients with T1D outweigh traditional risk factors for indicating the presence of diabetic retinopathy (DR).

## Research design and methods

We conducted a cross-sectional study with T1D patients recruited from the diabetes outpatient unit at the State University of Rio de Janeiro’s (UERJ) University Hospital. The local ethics committee approved the study, and written informed consent was obtained from all participants prior to enrollment. Regarding inclusion and exclusion criteria, T1D patients had to be ≥ 12 years old and have had diabetes for over one year. T1D was defined according to the American Diabetes Association criteria. Exclusion criteria included dermatological disease affecting the arms, use of tobacco or consumption of caffeine 24 h prior to the tests, undergoing physical exercise, and/or any hypoglycemic event on the morning of the tests.

Sample size was calculated based on an estimated prevalence of 40% of cardiovascular disease with a high CVR (RHI ≤ 1.67) considering a sensitivity of 80% and specificity of 85% of diagnostic tests with *p* value < 0.05 and with a confidence interval of 90%, indicating that at least 123 patients with T1D should be evaluated.

Patients underwent a demographic and clinical evaluation concerning age, self-reported color and race, age at diagnosis, diabetes duration, daily insulin doses, blood pressure, body mass index (BMI; kg/m^2^), self-reported frequency of physical activities (classified as sedentarism, less than three times a week, more than three times a week), and the use of drugs that could interfere with endothelial function, such as angiotensis-converting enzyme (ACE) inhibitors, statins, and angiotensin receptor blockers. Systolic and diastolic blood pressures (SBP and DBP, respectively) were measured twice at sites 1 min apart using an electronic sphygmomanometer (ONROM). The mean values were recorded as the patients’ clinical blood pressure, and the mean arterial pressure (MAP) was calculated as ${\text{DAP}}\,{ + }\,{1 \mathord{\left/ {\vphantom {1 3}} \right. \kern-\nulldelimiterspace} 3}\,\,\left( {{\text{SAP}} - {\text{DAP}}} \right)$.

Economic status was defined according to the Brazilian Economic Classification Criteria. This classification also considers education level, which is categorized as illiterate/incomplete primary education, complete primary education/incomplete secondary education, complete secondary education/incomplete high school, complete high school/some college, and complete college education. For this analysis, the following classes of economic status were considered: (1) high, (2) middle, (3) low, and (4) very low [17].

Venous blood samples were collected to assess laboratory parameters. Glycemic control was assessed by HbA1c (HPLC, Hitachi L-9100, Germany; reference range 2.6–6.2%). Capillary glycemia was determined using Abbott kits for the Optium glucometer (Abbott). Uric acid, serum creatinine, triglycerides, high-density lipoprotein (HDL) and total cholesterol (TC) levels were measured via enzymatic techniques (Cobas Mira; Roche, Bohemia, NY, USA). The normality values for uric acid levels ranged from 2.0 to 5.0 mg⁄dL. Low-density lipoprotein (LDL) cholesterol was calculated using Friedewald’s equation. Urinary albumin concentration was estimated via a solid-phase competitive chemiluminescent enzyme immunoassay (sensitivity of 0.5 l g⁄mL; Immulite 1000 Systems; DPC Medlab, Los Angeles, CA, USA), with intra- and inter-assay coefficients of variations at 4.4% and 6.1%, respectively. Serum C-reactive protein (CRP) was measured using a highly sensitive immunonephelometry assay (Behring Nephelometer; Behring, Germany) with a detection limit of 0.01 mg⁄dL and with intra- and inter-assay coefficients of variation at 1% and 5.3%, respectively.

sICAM-1 and sVCAM-1 were measured using multiplex assays (xMAP^®^ Technology) according to the manufacturer’s (Merck) instructions. sICAM-1 and sVCAM-1 were acquired in the MAGPIX^®^ equipment (LUMINEX^®^). We used the “Human Cardiovascular Disease panel 2 Magnetic Bead Panel Kit,” which includes bioassays performed on the surface of encoded fluorescent magnetic microspheres. The minimal detectable concentration and standard deviation were 0.019 (0.039) and 0.024 (0.048) for sICAM-1 and sVCAM-1, respectively.

### Evaluation of endothelial function by EndoPAT

EndoPAT was performed in the morning after a 20-min rest in the supine position in a temperature-controlled room (23 ± 1 °C) and approximately 1 h after a light breakfast. Microvascular reactivity and an RHI assessment through the EndoPAT 2000 device (Itamar Medical Ltd., Caesarea, Israel) are described elsewhere [18]. A manual sphygmomanometer cuff was placed on the forearm (study arm), and the other arm was used as control (control arm). After a 10-min baseline registration period, the forearm cuff was inflated to a pressure > 50 mm Hg SBP for 5 min. After rapid decompression, the device continuously captured changes in endothelium-dependent blood flow for 10 min. The software’s report displays the RHI value via EndoScore. It classifies CVR into three classes: (1) a score ≤ 1.67 indicates the need for immediate medical evaluation (high CVR); (2) a score between 1.68 and 2.0 indicates the endothelium is adequate, but appropriate preventive measures should be taken (intermediate CVR); and (3) a score ≥ 2.1 indicates the endothelium is intact, meaning low CVR (normal endothelial function). Information was provided by the manufacturer (Itamar-Medical Ltd., Ceaserea, Israel).

### Evaluation of renal function and retinopathy

Renal function was estimated using the chronic kidney disease epidemiology collaboration (CKD-EPI) equation [19] in adults and the Schwartz formula in adolescents [20] and was expressed as an estimated glomerular filtration rate (GFR) of mL/min per 1.73m^2^. No African American patients were considered in the CKD-EPI equation.

We screened DR via mydriatic binocular indirect ophthalmoscopy (BIO), which was performed by a retinal specialist who was trained before the beginning of the study at an ophthalmologic university center. The stage of retinopathy for each patient was considered based on the eye with more severe retinal impairment, and each eye was classified: (1) absent, (2) non-proliferative diabetic retinopathy, (3) proliferative diabetic retinopathy, or (4) macular edema according to the international classification of DR [21].

### Statistical analysis

First, an exploratory analysis expressed parametric data as mean values with standard deviations and nonparametric data as median values with interquartile ranges. A Mann–Whitney test was used to compare variables with nonparametric distribution and a T-test or analysis of variance (ANOVA) with Sidak correction was used for parametric distribution. We used Pearson’s univariate correlation when applicable. The frequency of physical activity was classified as patients without regular physical activity versus patients who exercised more than three times per week.

To further explore the relationship among biochemical markers (sVCAM-1 and sICAM-1), endothelial function (RHI), and use of drugs that could affect endothelial function (such as ACE inhibitors, statins, and angiotensin receptor blockers), and traditional risk factors related to DR (defined as present or absent), a multivariable hierarchical logistic regression (Backward Wald model) was used to determine which variables could be associated with the presence of DR (considered a dependent variable). To select the independent variables, we chose those with statistical significance in an exploratory or correlation analysis or those with clinical plausibility. Afterwards, the order of entry into the model was initially demographic and social data (age, gender, self-reported color-race, smoking, self-reported frequency of physical activity) followed by clinical data (BMI/abdominal circumference, SBP and DBP, and diabetes duration), laboratory data (HbA1c, uric acid, eGFR, and LDL-C) and finally endothelial function-related data (sVCM, sICAM, RIH, and use of drugs that may interfere with endothelial function).The model was assessed through a Hosmer–Lemeshow and Omnibus tests. The calculated Nagelkerke R^2^ and the odds ratio (OR) with a 95% confidence interval (CI) were expressed as indicated. A two-sided *P* value < 0.05 was considered statistically significant. The statistical analysis was performed using SPSS version 25.0.

## Results

### 1. ***Overview of the studied populaton***

**Fig. 1:**
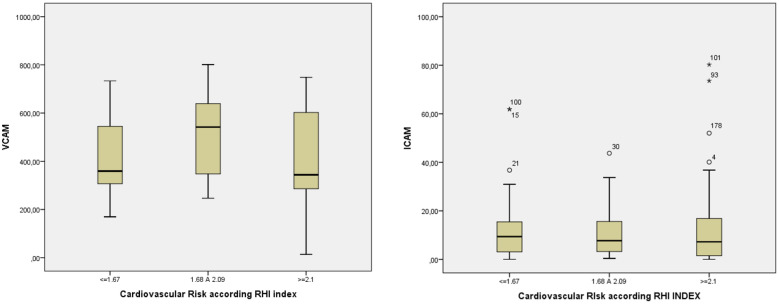
sVCAM-1 and sICAM-1 levels in patients with T1D according to Reactive Hyperemia Index (RHI). RHI ≤ 1.67 = higher cardiovascular risk; 1.67 < RHI < 2.1 = intermediate cardiovascular risk; RHI ≥ 2.1 = low cardiovascular risk

The present study included 187 patients with T1D. Overall, 56 (29.9%) patients had low CVR, 43 (23.0%) intermediate CVR, and 88 (47.1%) high CVR. In the pooled sample, different sVCAM-1 levels appeared (p = 0.01) when comparing the groups (stratified according to RHI) with low, medium, and high CVR. A significant difference appeared when comparing patients with a high CVR (RHI ≤ 1.67) to patients with an intermediate CVR (1.67 < RHI > 2.1) and when comparing patients with an intermediate CVR to patients with a low CVR (p = 0.01) as shown in Fig. 1.

Most patients presented non-proliferative DR 57 (30.5%), eight (4.3%) with proliferative DR and 16 (8.6%) with macular edema. Clinical and laboratory characteristics of the study’s population are shown in Table 1.

**Table 1:** Demographic, clinical and laboratory data of the studied population

Variables	N = 187
Female gender, n (%)	96 (51.3)
Age (years)	32.2 ± 13.9
Age at diagnosis (years)	15 [13]
Diabetes duration (years)	14 [15]
Daily insulin dose (U/Kg/day)	0.7 ± 0.3
Self-reported color-race, White, n (%)	95 (50.3)
Economic Status, n (%)	
Medium	25(13.4)
Low	132 (70.6)
Very Low	24(12.8)
Age at menarche (years)	12 .3 ± 1.6
Smoking, yes, n (%)	15 (8)
Exercise^a^, y n (%)	82 (43.9)
Abdominal circumference (women/men) (cm)	81 ± 11/ 82 ± 11
BMI (kg/m^2^)	24.2 ± 4.1
Systolic blood pressure (mmHg)	124 .7 ± 16.2
Diastolic blood pressure (mmHg)	74.8 ± 9.8
Pulse pressure (mmHg)	50.1 ± 12.4
Cardiac frequency (bpm)	78.1 ± 12.5

HbA1c (%/mmol/mol)	9.1 ± 2.1/ 76 ± 2.2
Uric acid (mg/dL)	3.8 ± 1.5
Total Cholesterol (mg/dL)	173.7 ± 38.8
Triglycerides (mg/dL)	70 [56]
HDL-cholesterol (mg/dL)	61.8 ± 18.5
LDL-cholesterol (mg/dL)	92.8 ± 28.8
CRP (mg/dL)	0.20 [0.43]

Use of endothelial interferent drug, n (%)	81 (43.3)
sVCAM-1 (ng/mL)	370.6 [277.7]
sICAM-1 (ng/mL)	8.3 [13.7]
RHI	1.7 [0.9]
Cardiovascular risk, according to RHI, n (%)	
High CVR; RHI ≤ 1.67	88 (47.1%)
Intermediate CVR, RHI 1.68–2.00	43 (23.0%)
Low CVR, RHI ≥ 2.1	56 (29.9%)
Chronic complications	
Retinopathy, y n (%)	81 (44.4)
Non-proliferative	57 (30.5)
Proliferative	8 (4.3)
Macular edema	16 (8.6)
eGFR ((mL/min/1.73 m^2^)	106.2 (28.2)

Data are expressed in mean ± standard deviation (SD) or median (Interquartil range, [IQR])

*IU* international units, *BMI* body mass índex, *HbA1c* glycated hemoglobin, *CRP* C reactive protein, *sVCAM-1* soluble vascular cytoadhesive molecule-1: (sVCAM-1), *sICAM-1* intracellular cytoadhesive molecule-1, *RHI* reactive hyperemia index, *CVR* cardiovascular risk, *eGFR* Glomerular filtration rate, *CKD* chronic renal disease

^a^Exercise ≥ 3 times/week

### 2. *Overview of the demographic, clinical, laboratory and endothelial function data according to the presence of diabetic retinopathy*

The presence of DR was associated with higher age, diabetes duration, lower frequency of self-reported physical activity, use of drugs that could affect endothelial function, BMI/abdominal circumference, SBP and DBP values, and lower levels of eGFR. A tendency toward higher levels of sVCAM was noted. No difference in, sICAM-1 and RHI levels appeared for these groups. These data are described in Table 2.

**Table 2:** Demographic, clinical, laboratorial and endothelial data stratified by the presence of diabetic retinopathy

Variables	Retinopathy				
	Absent n (%)		Present n (%)		p value
N	104 (55.6)		83 (44.4)		
Demographic data/clinical data					
Gender, female, n (%)	51 (49.0)		45 (54.2)		0.5
Age (years)	27.4 ± 13.3		38.1 ± 12.2		< 0.001
Age at diagnosis (years)	13.5 [11]		17 [15]		0.04
Diabetes duration (years)	10 [14]		21 [13]		< 0.001
Economic status, n (%)					0.7
Medium	16 (15.7)		9 (11.4)		
Low	73 (71.6)		59 (74.7)		
Very low	13 (12.7)		11 (13.9)		
Self-reported color-race, White, n (%)	50 (48.1)		44 (53.0)		0.3
Age at menarche (years)	12.4 ± 1.5		12.1 ± 1.7		0.3
Smoking, yes, n (%)	3 (2.9)		12 (14.5)		0.004
Exercise^a^, n (%)	55 (52.9)		27 (32.5)		0.004
Daily insulin dose (U/Kg/day)	0.69 ± 0.29		0.72 ± 0.3		0.3
Abdominal circumference, (cm)	74.8 ± 10.5		84.1 ± 11.8		0.01
BMI (kg/m^2^)	23.4 ± 3.8		24 .6 ± 4.7		0.07
Systolic blood pressure (mmHg)	119.9 ± 13.9		130.6 ± 16.9		< 0.001
Diastolic blood pressure (mmHg)	73.4 ± 9.1		76.5 ± 10.5		0.03
Mean blood pressure (mmHg)	89.0 ± 9.9		94.6 ± 11.3		0.001
Pulse pressure (mmHg)	46.8 ± 10.2		54.3 ± 13.6		< 0.001
Cardiac frequency (bpm)	77.2 ± 12.7		79.1 ± 12.3		0.3
Laboratorial data					
HbA1c (%/mmol/mol)	9.1 ± 2.1/76 ± 0.8		9.3 ± 2.2/78 ± 1.0		0.3
HbA1c ≤ 7.0%, N (%)	20 (10.7%)		7 (3.8%)		0.21
Uric acid (mg/dL)	3.6 ± 1.3		3.9 ± 1.6		0.12
Creatinine, mg/dL	0.75 [0.30]		0.79[0.34]		0.14
eGFR ((mL/min/1.73 m^2^)	117.0 ± 25.5		101.8 ± 26.9		< 0.001
Total Cholesterol (mg/dL)	174.5 ± 47.3		177.6 ± 35.8		0.6
Triglycerides (mg/dL)	71.5 [51]		78.0 [63]		0.8
HDL-cholesterol (mg/dL)	62.1 ± 20.7		61.1 ± 16.6		0.7
LDL-cholesterol (mg/dL)	91.1 ± 28.5		96.1 ± 27.8		0.2
CRP (mg/dL)	0.21 [0.4]		0.22 [0.46]		0.7
Endothelial data					
Use of drugs that interfere with the endothelium, n (%)	24 (12.8)		57 (30.4)		< 0.001
-Statins, n (%)	30 (21.4)		25 (53.2)		
-ACEi, n (%)	23 (16.4)		26 (55.3)		
-ARB, n (%)	5 (3.6)		5 (10.6)		
sVCAM-1 (ng/mL)	41 152.2		462.0 ± 167.8		0.08
sICAM-1 (ng/mL)	6.8 [11.5]		9.2 [14.6]		0.2
RHI	1.8 ± 0.66		1.92 ± 0.65		0.4
RHI, high CV risk; yes, n (%)	52 (50%)		36 (43.4)		0.5

Data are expressed in mean ± standard deviation (SD) or median Interquartil range, [IQR])

IU, international units, *BMI* body mass índex, *HbA1c* glycated hemoglobin, *eGFR* estimated glomerular filtration rate, *CRP* C reactive protein, *sVCAM-1* soluble vascular cytoadhesive molecule-1, *sICAM-1* soluble intracellular cytoadhesive molecule, *RHI* reactive hyperemia index, *CV* cardiovascular, *ACEi* angiotensin converting enzyme inhibitors, *ARB* angiotensin receptor blocker

^a^Exercise ≥ 3 times/week

Overall, 81 (43.3%) patients used drugs that could affect endothelial function. These patients were older (40.9 ± 11.8 versus 25.9 ± 12.0 years; p < 0.001) and had longer diabetes duration (20.7 ± 9.6 versus 10.9 ± 8.2 years; p < 0.001) than patients not using these drugs.

After stratifying the pooled group according to HbA1c levels (good, regular, or poor glycemic control if levels corresponded to ≤ 7.0%, 7.1 to 8.9% or ≥ 9.0%), 27 patients (14.4%) were classified as having good glycemic control. In this latter group, seven patients had retinopathy, and 20 did not. The comparison between the above-mentioned groups indicates that sICAM-1 levels were significantly higher in those patients with DR when compared with those without DR (15.47 ng/dL [4.6–46.7) versus 9.81 ng/dL [5.9–10.7]; p = 0.02]). sVCAM-1 levels did not show any differences between groups.

### 3. *Multivariable analysis with the presence of diabetic retinopathy as a dependent variable*

In the hierarchical multivariable logistic model, examining all selected independent factors that entered in the model, higher age, HbA1c, SBP and DBP, and higher frequency of drug use that could affect endothelial function were associated with DR in the final model. No association was noted concerning the sVCAM-1, sICAM-1, and RHI levels. All independent variables that were entered in the model could explain 36.4% (Nagelkerke R-squared) of a given patient having DR, and 72.3% of the patients were correctly classified by the model (Table 3).

**Table 3:** Final adjusted hierarchical multivariable logistic model with the presence of diabetic retinopathy as a dependent variable

	b	95% CI	p value
Age	0.040	1.008–1.075	0.014
Mean SBP	0.046	1.012–1.084	0.009
Mean DBP	− 0.046	0.907–1.006	0.082
HbA1c	0.269	1.085–1.580	0.005
Use of endothelial interfering drug	1.388	1,791–8.967	0.001

b: coefficient of logistic regression

*HbA1C* glycated hemoglobin, *95% CI* 95% confidence interval

## Discussion

The present study reveals that among patients with T1D, sVCAM-1, s-ICAM-1, and EndoPAT did not outweigh the traditional DR risk factors, such as age, blood pressure, and HbA1c, which were present in almost 45% of the patients. In the present study, the use of drugs that could affect endothelial function was also associated with DR.

Importantly, to the best of our knowledge, this study is the first one conducted in patients with T1D in which biochemical markers of ED and EndoPAT were evaluated; EndoPAT is a technique with an established correlation with coronary artery disease (CAD) that was validated in healthy non-diabetic subjects. So far, most studies that used EndoPAT involved patients with T2D who were generally older and had more comorbidities (such as hypertension and CAD) than did the patients in our study were [14, 22].

T1D is associated with a two to tenfold higher risk of mortality and cardiovascular disease due to the development of comorbidities [23]. Functional and anatomical impairment of endothelial functions apparently impact small and large vascular beds already during the onset of diabetes, many years before the development of vascular complications [6]. A recent meta-analysis concerning the prevalence of ED found that both endothelium-dependent vasodilation (endothelial function) and non-endothelium-dependent vasodilation (vascular smooth muscle function) was over 35% in individuals within five years after the diagnosis [24].

Soluble adhesion molecules (sICAM-1, sVCAM-1) could be surrogate biochemical markers of ED [8]. For T1D, however, studies have shown conflicting results in terms of use of these markers [25–28]. Compared to healthy patients, Fasching et al. [25] demonstrated increased levels of both molecules and a significant association between sVCAM-1 levels and microvascular complications in patients with T1D. This study also showed an association between these biochemical markers and subclinical inflammation in individuals with T1D diagnosed before 36 years of age. However, Ugurlu et al. evaluated 59 T1D patients using biochemical markers (sVCAM-1 and sICAM-1) and flow-mediated dilation of endothelium and did not find differences between groups with/without DR [26]. In the present study, only sVCAM-1 was considered a possible biomarker of inflammatory activity because we found lower levels in the group with the lowest CVR (according to RHI) out of the whole sample. Unexpectedly, higher levels appeared in the group with an intermediate CVR who did not use endothelial-interfering drugs. This difference could be attributed to the use of more endothelial interfering drugs by our sample’s patients with microvascular complications, including DR, than in those without complications; thus, ED and consequently, RHI, were ameliorated.

Although sICAM levels were significantly higher in the presence of DR among those patients with good glycemic control as demonstrated in the univariate analysis, this finding did not persist after adjustments were made in the multivariate analysis. However, the presence of higher levels of sICAM in those with DR can be explained by the fact that diabetes increases the level of ICAM-1 on the luminal surface of endothelial cells in the retina when compared with other tissues as described in a recently published experimental study [29]. However, our findings contrasted those of Fathollahi et al. a comparison of HbA1c levels of the T1D patients with the plasma levels of sICAM-1, sVCAM-1, and sE-selectin in those with/without microvascular complications that did not yield any significant correlation between these molecules and glycemic control or microvascular complications [30].

Nevertheless, sVCAM-1 was a possible biomarker of ED in our patients with T1D based on an exploratory analysis. After adjustments, the most important factors associated with DR in our sample were the standard risk factors: (1) HbA1c, (2) blood pressure, and the use of drugs that could interfere with endothelial function. Our findings are similar to a Brazilian nested case control study in which it was found that out of interleukin 6 (IL-6), CRP, tumor necrosis alpha (TNF⍺), and vascular endothelial growth factor (VEGF) serum levels among patients with T1D with/without DR, CRP was the only biomarker associated with DR. Thus, glycemic control, use of ACE inhibitors, and renal function appear to be the most significant clinical variables presenting as risk factors for DR [31]. However, the findings of the present study do not agree with those found by Soedamah-Muthu et al., who demonstrated a positive association between sVCAM-1 levels and microvascular complications, such as retinopathy, nephropathy, and CVD [32]. However, patients from this latter study were older and had longer diabetes durations than our patients. Considering the data described in these different studies [31, 32], we can conclude that so far it is difficult to establish a relationship between serum levels of ED biochemical and inflammatory markers with the levels of these biomarkers in retinal endothelium and vitreous body.

Subtle impairment of retinal microcirculatory system due to arteriolar widening, greater tortuosity, and venular dilation precedes clinical retinopathy [33]. However, the pro-inflammatory biomarkers studied in our sample disclosed no association with DR. One possible explanation for this lack of association is the fact that biochemical mediators responsible for microvascular ED need to be clarified, especially in the presence of other known risk factors, such as hypertension and hyperglycemia, which affect smaller vessels and are well-established DR risk factors [33].

The strength of our study is the evaluation of patients with T1D from an admixed population who were screened for ED using two methods: (1) EndoPAT and (2) the biochemical markers sVCAM-1 and sICAM-1 for the presence of DR.

Finally, we must address some study limitations. An important limitation is the use of endothelial interfering drugs that could not be discontinued for medical and ethical reasons. In addition, the lack of studies performed with patients with T1D with this methodology makes comparisons difficult. Moreover, this cross-sectional study with only a population of patients with T1D does not allow us to infer any causal relationship. Another limitation is the classification of DR into two categories (absent or present) for statistical purposes due to the number of cases in each degree of DR. Furthermore, Endo-PAT’s RHI used to distinguish cardiovascular risk levels were extrapolated from data described in T2D patients due to the lack of scientific results showing its accuracy in patients with T1D. Another limitation is that other endothelial microparticles, such as Von Willebrand factor, soluble E-selectin, and soluble thrombomodulin were not assessed in this study, a study of which could have enhanced our knowledge of vascular biology.

In conclusion, in our sample, serum biomarkers and microvascular reactivity did not outweigh the traditional DR risk factors, such as age, HbA1c, arterial blood pressure and use of drugs that could interfere with endothelial function and were truly the factors significantly associated with DR. Further prospective studies must assess the strength of endothelial function markers as predictors of diabetes-related micro- and macro-vascular chronic complications in patients with T1D.

## Data Availability

The datasets used and/or analysed during the current study are available from the corresponding author on reasonable request.

## Abbreviations

ACh: Acetylcholine
ACE: Angiotensin-converting enzyme
AGEP: Advanced glycosilation end products
BMI: Body mass index
CVD: Cardiovascular disease
CVR: Cardiovascular risk
CKD: Chronic kidney disease
DM: Diabetes mellitus
DBP: Diastolic blood pressure
DR: Diabetic retinopathy
ED: Endothelial dysfunction
GFR: Glomerular flow rate
MAP: Mean arterial pressure
T1D: Type 1 diabetes
RHI: Reactive hyperemia index
SAP: Systolic arterial pressure
sVCAM-1: Soluble vascular cytoadhesive molecule-1 (sVCAM-1)
s-ICAM-1: Intracellular cytoadhesive molecule-1
